# Malate-Aspartate Shuttle Plays an Important Role in LPS-Induced Neuroinflammation of Mice Due to its Effect on STAT3 Phosphorylation

**DOI:** 10.3389/fmolb.2021.655687

**Published:** 2021-07-26

**Authors:** Cuiyan Zhou, Wangsong Shang, Shan-Kai Yin, Haibo Shi, Weihai Ying

**Affiliations:** ^1^ School of Biomedical Engineering and Med-X Research Institute, Shanghai Jiao Tong University, Shanghai, China; ^2^ Department of Otorhinolaryngology, Shanghai Sixth People’s Hospital Affiliated to Shanghai Jiao Tong University, Shanghai, China

**Keywords:** malate-aspartate shuttle, neuroinflammation, aminooxyacetic acid, pyruvate, stat3

## Abstract

Neuroinflammation is a key pathological factor in numerous neurological disorders. Cumulating evidence has indicated critical roles of NAD^+^/NADH metabolism in multiple major diseases, while the role of malate-aspartate shuttle (MAS) - a major NADH shuttle - in inflammation has remained unclear. In this study we investigated the roles of MAS in LPS-induced neuroinflammation both *in vivo* and *in vitro*. Immunofluorescence staining, Western blot assay and Real-time PCR assays were conducted to determine the activation of Iba-1, the protein levels of iNOS and COX2 and the mRNA levels of IL-1β, IL-6, and TNF-α *in vivo*, showing that both pre-treatment and post-treatment of aminooxyacetic acid (AOAA) - an MAS inhibitor - profoundly decreased the LPS-induced neuroinflammation in mice. BV2 microglia was also used as a cellular model to investigate the mechanisms of this finding, in which such assays as Western blot assay and nitrite assay. Our study further indicated that AOAA produced its effects on LPS-induced microglial activation by its effects on MAS: Pyruvate treatment reversed the effects of AOAA on the cytosolic NAD^+^/NADH ratio, which also restored the LPS-induced activation of the AOAA-treated microglia. Moreover, the lactate dehydrogenase (LDH) inhibitor GSK2837808A blocked the effects of pyruvate on the AOAA-produced decreases in both the cytosolic NAD^+^/NADH ratio and LPS-induced microglial activation. Our study has further suggested that AOAA produced inhibition of LPS-induced microglial activation at least partially by decreasing STAT3 phosphorylation. Collectively, our findings have indicated AOAA as a new and effective drug for inhibiting LPS-induced neuroinflammation. Our study has also indicated that MAS is a novel mediator of LPS-induced neuroinflammation due to its capacity to modulate LPS-induced STAT3 phosphorylation, which has further highlighted a critical role of NAD^+^/NADH metabolism in inflammation.

## Introduction

Neuroinflammation is a key pathological factor in multiple major neurological disorders including cerebral ischemia, Alzheimer’s disease (AD) and Parkinson’s disease (PD) (Hirsch and Hunot 2009; Perry et al., 2010). Microglial activation plays a crucial role in the inflammatory responses in the brain, which can induce brain damage by generating cytokines and oxidative stress (Perry et al., 2010). Therefore, it is of great significance to further investigate the regulatory mechanisms of microglial activation. Signal transducer and activator of transcription 3 (STAT3) - a member of STAT family, has been found to be a mediator of inflammatory response (Hu et al., 2019): Inhibition of STAT3 phosphorylation can mitigate inflammatory responses (Qin et al., 2016; Yang et al., 2019), including LPS-induced microglial activation (Qin et al., 2017; Liu et al., 2019).

Cumulating evidence has indicated that NAD^+^/NADH metabolism plays crucial roles in both aging and numerous diseases: Multiple studies have found significant decreases in NAD^+^ levels in the models of aging and a number of diseases (Ying 2008; Verdin 2015). NAD^+^ administration can also produce profound beneficial effects in animal models of aging and multiple diseases (Ying et al., 2007; Yoshino et al., 2011; Mills et al., 2016; Das et al., 2018). However, there has been significant deficiency in the information regarding the roles of NAD^+^/NADH metabolism in inflammation. Since inflammation is a key pathological factor in numerous diseases, it is warranted to elucidate the relationships between NAD^+^/NADH metabolism and inflammation.

NADH shuttle is one of the major systems that regulate cellular NAD^+^/NADH metabolism (Ying 2008): NADH shuttle mediates the transfer of the reducing equivalents of cytosolic NADH into mitochondria (Satrustegui and Bak 2015). While NADH shuttles include malate-aspartate shuttle (MAS) and glycerol-3-phosphate shuttle (Ying 2008), MAS is the major NADH shuttle in the brain (McKenna et al., 2006). Aspartate aminotransferase (AST), a pyridoxal phosphate (PLP)-dependent transaminase, is the rate-limiting enzyme of MAS (Fitzpatrick et al., 1983). AOAA is a most widely used inhibitor of MAS, which inhibits MAS by targeting at AST (McKenna et al., 2006; Jespersen et al., 2017).

Our previous study has found that AOAA can significantly decrease LPS-induced activation of BV2 microglia (Shang et al., 2017), suggesting that MAS may be involved in LPS-induced microglial activation. However, it remains unknown if AOAA can decrease LPS-induced neuroinflammation *in vivo*. Moreover, considering that AOAA can also inhibit cystathionine-β-synthase (CBS), a major enzyme for endogenous H_2_S production (Qu et al., 2013; Zhao et al., 2017; Spalloni et al., 2019), it remains to be determined if AOAA produces its inhibitory effects on LPS-induced microglial activation mainly by its effects on MAS.

Based on our previous observations, two major goals of our current study were established: First, to determine if AOAA can decrease LPS-induced neuroinflammation *in vivo*; and second, to determine if AOAA produces its inhibitory effects on LPS-induced microglial activation by its effects on MAS. By using LPS-induced neuroinflammation of mice and LPS-induced activation of microglial cell cultures as animal and cellular models of neuroinflammation, our study has indicated AOAA as a new and effective drug for inhibiting LPS-induced neuroinflammation. Our findings have also indicated MAS as a novel mediator of LPS-induced neuroinflammation due to its capacity to modulate LPS-induced STAT3 phosphorylation.

## Materials and Methods

### Reagents

AOAA (C13408), LPS (L2880), Pyruvate (P4562) were purchased from Sigma Aldrich (St Louis, MO, United States). GSK2837808A was purchased from MedChemExpress (New Jersey, United States). Primers were purchased from Sangon Biotech (Shanghai, China).

### Cell Cultures

BV2 microglia were plated into 24-well or 12-well cell culture plates at the initial density of 1 × 10^5^ cells/ml in Dulbecco’s Modified Eagle Medium containing 4,500 mg/L D-glucose, 584 mg/L L-glutamine (Thermo Scientific, Waltham, MA, United States), 1% penicillin and streptomycin (Invitrogen, Carlsbad, CA, United States), supplemented with 10% heat-inactivated (56°C for 30 min) fetal bovine serum (Gibco, Melbourne, Austria). The cells were maintained at 37°C in a 5% CO_2_ incubator.

### Animal Studies

Animal research in this study was conducted according to an animal protocol approved by the Ethics Committee of Animal Study of the School of Biomedical Engineering, Shanghai Jiao Tong University (Approval#: 20111001). Male wild-type C57BL/6 mice aged 5–6 weeks were purchased from Shanghai Jiesijie Laboratory Animal Inc. (Shanghai, China). Mice were allowed free access to food and water and housed in a 12 h of light and dark per day at an ambient temperature of 22°C. 2 weeks later, the animals were randomized into four groups: (1) The control group (*N =* 10): The mice were intraperitoneally (i.p.) administered with PBS daily for 10 days. (2) The AOAA group (*N =* 10): The mice were i.p. administered with 10 mg/kg AOAA daily for 10 days. The dosages of AOAA were determined according to previously published studies (Ahmad and Szabo 2016; Blancquaert et al., 2016; Luo et al., 2019; Zhao et al., 2020). (3) The LPS group (*N =* 10): The mice were i.p. administered with PBS for 3 days and subsequently injected with 250 μg/kg LPS each day for another 7 days. This model was established on the basis of the information from several previous studies, which reported that i.p. administration with 250 μg/kg LPS for 1 week induced significant neuroinflammatory responses in C57 mice (Badshah et al., 2016; Chen et al., 2019; Guan et al., 2019). (4) The AOAA pre-treatment group (*N =* 10): The mice were i.p. administered with 10 mg/kg AOAA daily for 3 days and subsequently injected with 250 μg/kg LPS each day for 7 days. (5) The AOAA post-treatment group (*N =* 10): After the mice were i.p. administered with PBS for 3 days and subsequently i.p. administered with 250 μg/kg LPS for 3 days, the mice were injected with both 250 μg/kg LPS and 10 mg/kg AOAA for another 4 days. The mice were i.p. administered with tribromoethanol and then perfused with PBS and 4% paraformaldehyde (PFA) solution (for immunofluorescence staining assay), and the brain tissues were flash-frozen and sliced with 30-μm thickness using a cryostat or extracted for Western blot assays or real-time PCR assays.

### Cytosolic NAD^+^/NADH Ratio Assay

Cyttosolic NAD^+^/NADH ratio was determined by measuring pyruvate/lactate levels in cell cytoplasm (20): The cell cultures were treated with different drugs. After the drug treatment, the cells were washed 3 times with cold PBS and extracted with 200 μl 0.5 N perchloric acid (PCA). After centrifugation at 12,000 rpm for 10 min at 4°C, the supernatant was neutralized to pH 6.7 using 3 N KHCO_3_. After centrifugation at 12,000 rpm for 5 min at 4°C, 40 µl supernatant and standards were pipetted into a 96-well black plate, and mixed with 80 µl reaction medium containing 0.11 unit/ml pyruvate oxidase (Sigma Aldrich, St Louis, MO, United States), 37 µM Amplex Red (Molecular Probes, Eugene, OR, United States) and 0.15 unit/ml horseradish peroxidase (HRP) (Sigma Aldrich, St Louis, MO, United States) in pyruvate assay buffer (pH 6.7) for pyruvate assay. 50 µl supernatant and standards were pipetted into a 96-well black plate, and mixed with 50 µl reaction medium containing 0.25 unit/ml lactate oxidase (Sigma Aldrich, St Louis, MO, United States), 50 µM Amplex Red (Molecular Probes, Eugene, OR, United States) and 0.4 unit/ml HRP (Sigma Aldrich, St Louis, MO, United States) in 50 mM sodium phosphate for lactate assay. After 15 min (for pyruvate assay) or 30 min (for lactate assay), fluorescence intensity was determined by a plate reader (Synergy 2, BioTek), with excitation wavelength of 535 nm and emission wavelength of 590 nm.

### Nitrite Production Assay

The nitric oxide (NO) levels in the culture media of BV2 microglia were determined by the Griess reaction using a commercially available kit (Beyotime Institute of Biotechnology, Jiangsu, China). The assay was performed using a plate reader according to the manufacturer’s protocol. Briefly, 50 μl of medium of sample was mixed with 50 μl of Griess reagent I and Griess reagent II in a 96-well plate. Subsequently the A_540 nm_ of the samples was determined.

### Western Blot Assay

BV2 cells and the hippocampus or cortex of mice were harvested and lysed in RIPA buffer (Millipore, Temecula, California, United States) containing 1% protease inhibitor cocktail, 1% phosphatase inhibitor cocktail (CWBio, Beijing, China) and 1 mM phenylmethanesulfonyl fluoride. The lysates were centrifuged at 12,000 rpm for 10 min at 4°C. After quantifications of the protein samples using BCA Protein Assay Kit (Pierce Biotechnology, Rockford, Illinois, United States), 30 μg of total protein was electrophoresed through a 10% sodium dodecyl sulfate - polyacrylamide gel and then transferred to 0.45-μm nitrocellulose membranes. The blots were incubated overnight at 4°C with primary antibodies. The antibody dilutions were as follows: anti-iNOS antibody (1:1,000 dilution, Abcam, Cambridge, United States), anti-COX2 antibody (1:800 dilution, Abcam, Cambridge, United States), anti-p-STAT3 antibody (1:2,000 dilution, Cell Signaling Technology, Danvers, MA), anti-STAT3 antibody (1:2,000 dilution, Cell Signaling Technology, Danvers, MA) anti-Actin antibody (1:1,000 dilution, Santa Cruz, California, United States). Subsequently the membranes were incubated with horse radish peroxidase-conjugated secondary antibody (1:3,000 dilution, Epitomics, Hangzhou, China) in TBST containing 1% BSA at room temperature for 1 h. The intensities of the bands were quantified by densitometry using Gel-Pro Analyzer (Media Cybernetics, Silver Spring, Maryland, United States).

### Real-Time PCR Assays

For mRNA quantification, total RNA from brain tissue was extracted by using RNA extraction kits (BioTeke Corporation, Beijing, China) and was quantified by NanoDrop spectrophotometer (ND-2000, NanoDrop Technologies, United States). For reverse transcription, the total RNA of each sample (500 nanograms) was converted into cDNA using PrimeScript RT Master Mix (TaKaRa, Dalian, China). After cDNA synthesis, Real-time PCR was performed with SYBR Premix Ex Taq (TaKaRa, Dalian, China) following the manufacturer’s protocol. The mRNA expression of IL-1β, IL-6, and TNF-α was normalized to GAPDH. The primer sequences used were listed as following: IL-1β, (forward) 5’-AAG GGCTGCFTTCCAAACCTTTGAC-3’, (reverse)5’-ATACTGCCTGCCTGAAGCTCTTGT-3’; IL-6, (forward)5’-TCCATCCAGTTGCCTTCTTG-3’, (reverse)5’-AAGCCTCCGACTTGTGAAGTG-3’; TNF-α, (forward)5’-CCCTCACACTCAGATCATCTTCT-3’, (reverse)5’-GCTACGACGTGGGCTA CAG-3’; GAPDH, (forward)5’-CCCTGCACCACCAACTGCTTA-3’, (reverse)5’-GGCCATCCACAGTCTTCT GA -3’.

### Immunofluorescence Staining

Brain cryosections were fixed in 4% PFA for 15 min, followed by incubation with 0.2% Triton X-100 in PBS for 10 min. After washes with PBS three times, the sections were blocked with 10% goat serum for 1 h at room temperature and then incubated with Iba-1 antibody (1:500 dilution, Wako, Osaka, Japan) or COX2 antibody (1:200 dilution, Abcam) in PBS containing 1% goat serum overnight at 4°C. After three washes with PBS, the sections were incubated with Alexa Fluor® 488 goat anti-rabbit IgG (H + L) Secondary antibody (1:500 dilution, Molecular Probes, Eugene, Oregon, United States) in PBS containing 1% goat serum for 1 h at room temperature. After three washes with PBS, the sections were counterstained with DAPI (1:1,000 dilution, Beyotime Institute of Biotechnology) in PBS for 5 min at room temperature. The fluorescence images were photographed using a Leica confocal microscope (Leica Microsystems, Wetzlar, Germany), which were analyzed by ImageJ.

### Statistical Analyses

All data are presented as mean ± SEM. Data were assessed by one-way ANOVA, followed by Student-Newman-Keuls post hoc test. *p* values less than 0.05 were considered statistically significant.

## Results

### AOAA Administration Profoundly Decreased LPS-Induced Neuroinflammation of C57BL/6 Mice

**FIGURE 1:**
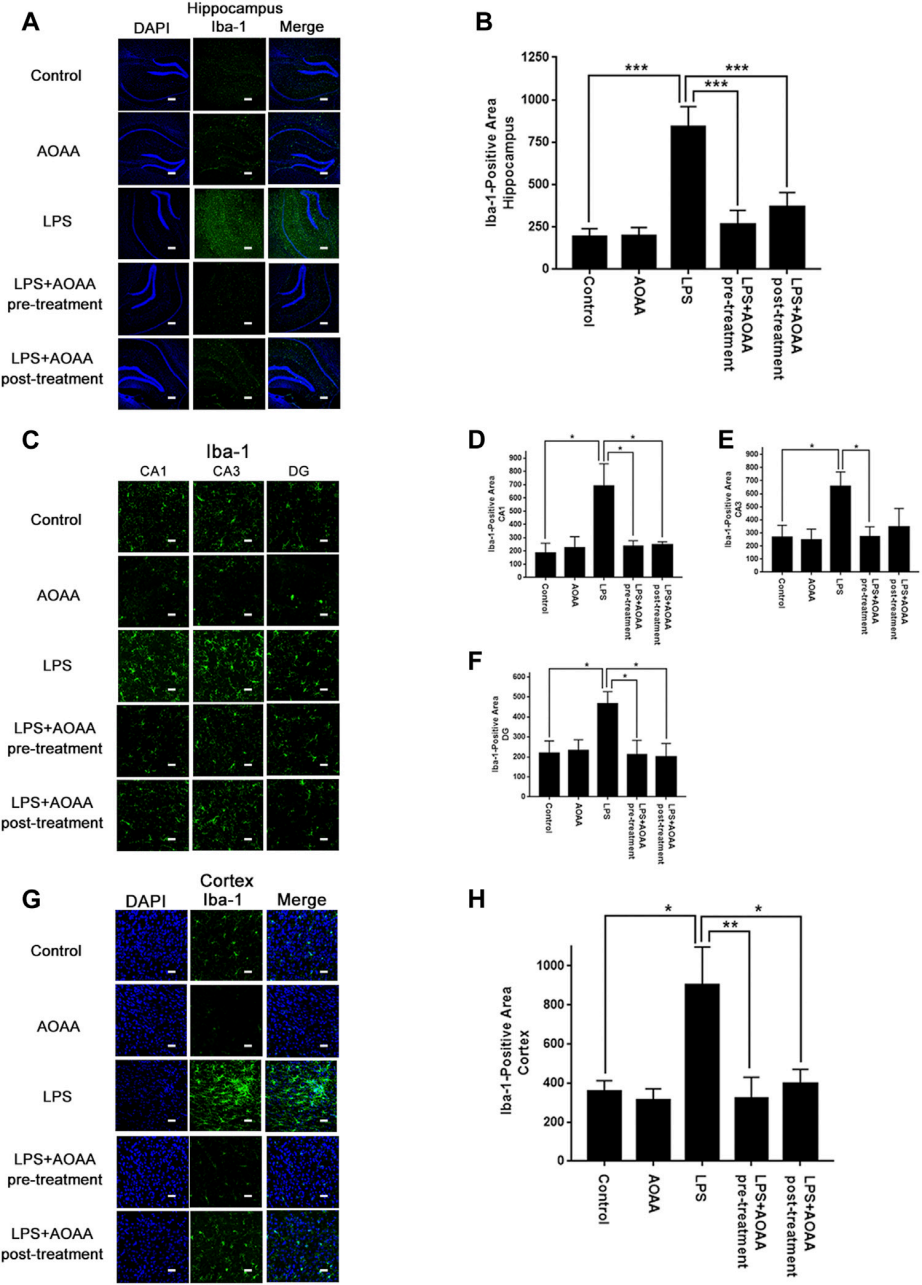
Both AOAA pre-treatment and post-treatment attenuated LPS-induced Iba-1 increases in the hippocampus and the cerebral cortex of C57BL/6 mice. **(A)** The immunofluorescence staining assay showed that both pre-treatment and post-treatment of AOAA decreased LPS-induced Iba-1 increases in the hippocampus of the mice. Scale bar = 100 μm. **(B)** Quantifications of the images indicated that both pre-treatment and post-treatment of AOAA significantly decreased LPS-induced Iba-1 increases in the hippocampus of the mice. **(C)** The immunofluorescence staining assay showed that both pre-treatment and post-treatment of AOAA decreased LPS-induced Iba-1 increases in the hippocampal CA1, CA3, DG areas of the mice. Scale bar = 20 μm. **(D–F)** Quantifications of the images indicated that both pre-treatment and post-treatment of AOAA significantly decreased LPS-induced Iba-1 increases in the hippocampal CA1, CA3, DG areas of the mice. **(G)** The immunofluorescence staining assay showed that both pre-treatment and post-treatment of AOAA decreased LPS-induced Iba-1 increases in the cerebral cortex of the mice. Scale bar = 20 μm. **(H)** Quantifications of the images indicated that both pre-treatment and post-treatment of AOAA significantly decreased LPS-induced Iba-1 increases in the cerebral cortex of the mice. The mice were administered with LPS (250 μg/kg, i.p,). AOAA was administered 3 days before LPS administration or 3 days after LPS administration. 7 days after the LPS administration, immunofluorescence staining assays were conducted. For each animal, three slices of the brain were used for the analyses. The mean of the data from analyses of the three slides was used for statistical analysis. *N* = 5. *, *p* < 0.05, ***, *p* < 0.001.

We determined the effects of AOAA on LPS-induced neuroinflammation of mice by assessing the effects of AOAA on three major hallmarks of neuroinflammation, including increased Iba-1 levels - a hallmark of microglial activation (Nam et al., 2018; Zeng et al., 2019), increased protein levels of iNOS and COX2, as well as increased levels of IL-1β, IL-6 and TNF-α mRNA. We found that LPS induced significant increases in Iba-1 in the hippocampal CA1, CA3, and DG areas (Figures 1A,C), as well as the cerebral cortex (Figure 1G), all of which were abolished by pre-treatment of 10 mg/kg AOAA (Figures 1B,D–F,H). Post-treatment of AOAA also abolished the LPS-induced Iba-1 increases in all of the examined brain regions, except the hippocampal CA3 area (Figures 1B,D–F,H).

**FIGURE 2:**
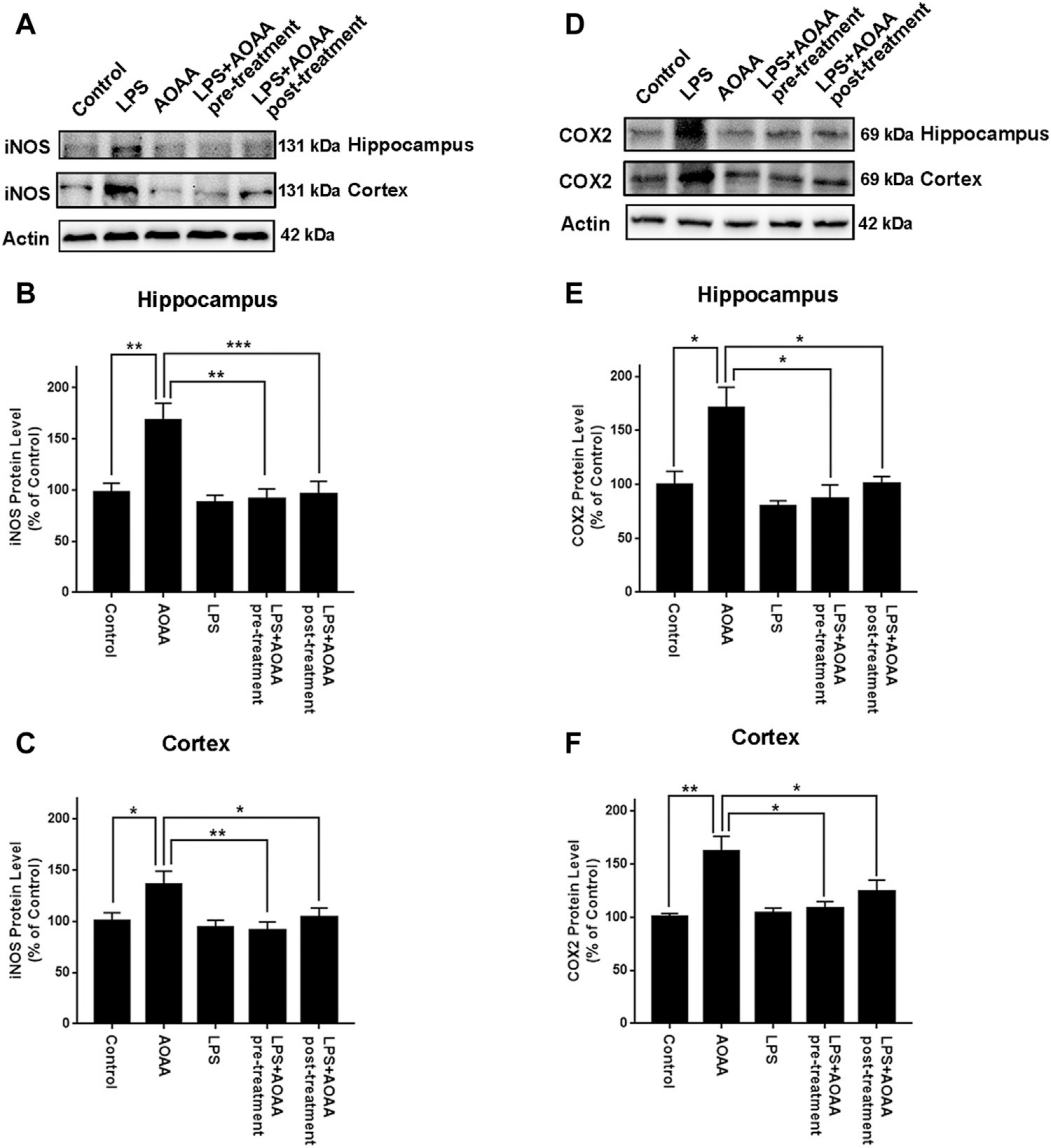
Both AOAA pre-treatment and post-treatment inhibited LPS-induced increases in the protein levels of iNOS and COX2 in the hippocampus and the cerebral cortex of C57BL/6 mice. **(A)** Western blot assays showed that both pre-treatment and post-treatment of AOAA attenuated LPS-induced increase in the iNOS protein levels in the hippocampus and the cerebral cortex of mice. **(B, C)** Quantifications of the Western blots showed that both pre-treatment and post-treatment of AOAA significantly attenuated LPS-induced increase in the iNOS protein levels in the hippocampus and the cerebral cortex of mice. **(D)** Western blot assays showed that both pre-treatment and post-treatment of AOAA attenuated LPS-induced increase in the COX2 protein levels in the hippocampus and the cerebral cortex of mice. **(E, F)** Quantifications of the Western blots showed that both pre-treatment and post-treatment of AOAA significantly attenuated LPS-induced increase in the COX2 protein levels in the hippocampus and the cerebral cortex of mice. The mice were administered with LPS (250 μg/kg, i.p,). AOAA was administered 3 days before LPS administration or 3 days after LPS administration. 7 days after the LPS administration, Western blot assays were conducted. *N* = 5. *, *p* < 0.05, **, *p* < 0.01; ***, *p* < 0.001.

We also found that LPS induced significant increases in the protein levels of iNOS in the hippocampus and the cerebral cortex, which were abolished by both pre-treatment and post-treatment of 10 mg/kg AOAA (Figures 2A–C). Similarly, LPS induced significant increases in the protein levels of COX2 in the hippocampus and the cerebral cortex, which were also abolished by both pre-treatment and post-treatment of 10 mg/kg AOAA (Figures 2D–F). Immunofluorescence staining assays indicated that both pre-treatment and post-treatment of AOAA markedly attenuated the LPS-induced increases in the COX2 signal in the hippocampus and the cerebral cortex (Supplementary Figures 1A–D).

**FIGURE 3:**
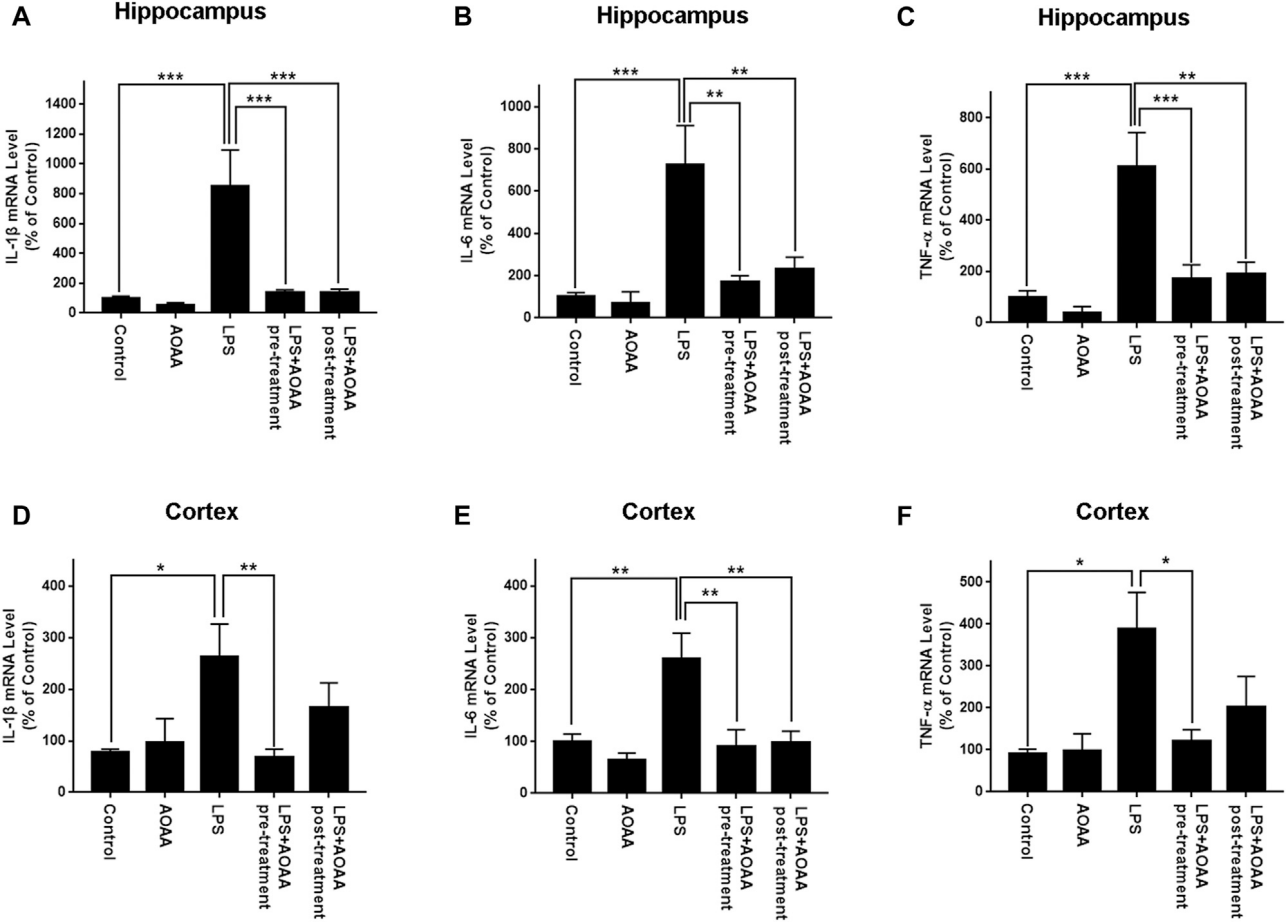
Real-time PCR assays showed that AOAA significantly attenuated LPS-induced increases in the levels of IL-1β, IL-6, TNF-α mRNA in the hippocampus and the cerebral cortex of C57BL/6 mice. **(A–C)** Real-time PCR assays showed that both AOAA (10 mg/kg, i.p.) pre-treatment and post-treatment significantly attenuated LPS-induced increases in the levels of IL-1β, IL-6, TNF-α mRNA in the hippocampus of the mice. **(D–F)** Real-time PCR assays showed that both AOAA (10 mg/kg, i.p.) pre-treatment and post-treatment significantly attenuated LPS-induced increases in the levels of IL-1β, IL-6, TNF-α mRNA in the cerebral cortex of the mice. The mice were administered with LPS (250 μg/kg, i.p,). AOAA was administered 3 days before LPS administration or 3 days after LPS administration. 7 days after the LPS administration, Real-time PCR assays were conducted. *N* = 5. *, *p* < 0.05, **, *p* < 0.01.

Real-time PCR assays were conducted to determine the effects of AOAA on LPS-induced increases in the levels of IL-1β, IL-6, TNF-α mRNA in the brain. LPS induced significant increases in the mRNA levels of these three cytokines in both hippocampus and cerebral cortex of the mice (Figures 3A–F). All of these mRNA increases were abolished by pre-treatment of 10 mg/kg AOAA. Post-treatment of AOAA also significantly attenuated the LPS-induced increases in the mRNA levels of IL-1β (Figure 3A) and IL-6 (Figure 3B) in the hippocampus, as well as the LPS-induced increase in the mRNA level of IL-6 in the cerebral cortex (Figure 3E). We also found that the LPS treatment produced a significant decrease in the body weight of the mice in the first three days after the LPS treatment. Subsequently the body weight of the mice was gradually recovered (Supplementary Figures 2C–E). Neither saline nor AOAA significantly affected the body weight of the mice (Supplementary Figures 2A–B).

### Pyruvate Reversed the Inhibitory Effects of AOAA on LPS-Induced Activation of BV2 Microglia by Promoting the LDH-Mediated Monsumption of Cytosolic NADH

**FIGURE 4:**
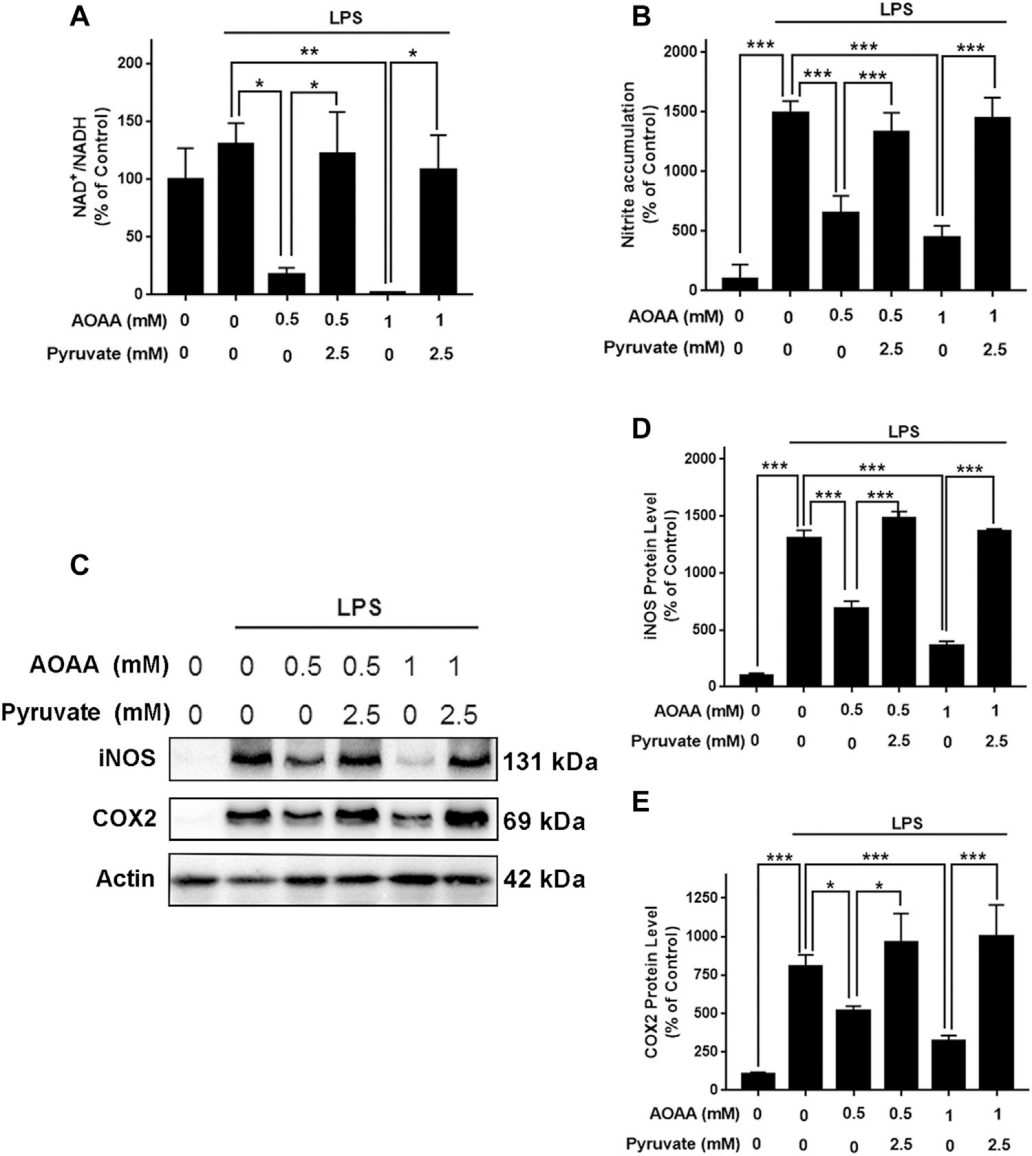
Pyruvate treatment reversed the effects of AOAA on the cytosolic NAD^+^/NADH ratio and microglial activation of LPS-treated BV2 microglia. **(A)** Treatment of the cells with 2.5 mM pyruvate completely reversed the AOAA-produced decrease in the cytosolic NAD^+^/NADH ratio of the LPS-treated BV2 microglia. **(B)** Pyruvate virtually abolished the AOAA-produced inhibition of the LPS-induced increases in the intracellular NO levels of the cells. **(C)** Western blot assays showed that pyruvate attenuated the AOAA-produced inhibition of the LPS-induced increases in the protein levels of iNOS and COX2 in the cells. **(D)** Quantifications of the Western blot assays showed that pyruvate significantly attenuated the AOAA-produced inhibition of the LPS-induced increases in the protein levels of iNOS in the microglial cell cultures. **(E)** Quantifications of the Western blot assays showed that pyruvate significantly attenuated the AOAA-produced inhibition of the LPS-induced increases in the protein levels of COX2 in the microglial cell cultures. After pre-treatment with 0.5 or 1 mM AOAA for 30 min, the BV2 cells were incubated with 2.5 mM pyruvate and 1 µg/ml LPS for 20 h (for NAD^+^/NADH assay) or 23.5 h (for NO, iNOS, and COX2 assays). *N* = 3. The data were collected from three independent experiments. *, *p* < 0.05, **, *p* < 0.01; ***, *p* < 0.001.

To determine if AOAA produces its inhibitory effects on LPS-induced microglial activation by its effects on MAS, we applied pyruvate and LDH inhibitors in our study based on the following considerations: A key and direct effect of MAS inhibition is increased accumulation of cytosolic NADH thus leading to decreased cytosolic NAD^+^/NADH ratios. It has been reported that exogenous pyruvate is completely converted to lactate by LDH rather than oxidized in the TCA cycle (Hereng et al., 2011), which can increase LDH-mediated consumption of cytosolic NADH leading to increase cytosolic NAD^+^/NADH ratios. Therefore, we planned to determine if pyruvate can reverse the effect of AOAA on LPS-induced microglial activation. Furthermore, we determined if LDH inhibitors can block this effect of pyruvate. Concentration-dependent experiments were conducted to determine the optimal concentrations of LPS, AOAA and pyruvate which would be used in our experiments, showing that 1 μg/ml LPS, 0.5 and 1 mM AOAA, as well as 2.5 mM pyruvate could be used in our study: Maximal activation of BV2 microglia was produced by 1 μg/ml LPS (Supplementary Figure 3A); both 0.5 and 1 mM AOAA produced significant inhibition of the LPS-induced microglial activation (Supplementary Figure 3B) and 2.5 mM pyruvate reversed the effect of AOAA (Supplementary Figure 3D). Both 0.5 or 1 mM AOAA and 2.5 mM pyruvate did not produce cytotoxicity (Supplementary Figures 3C,E). Our study showed that AOAA produced a significant decrease in the cytosolic NAD^+^/NADH ratio of BV2 microglia, which was largely reversed by pyruvate (Figure 4A). Pyruvate also virtually abolished the AOAA-produced inhibition of the LPS-induced microglial activation, as indicated by its effects on the intracellular NO levels and the protein levels of iNOS and COX2 in the microglia treated with LPS and AOAA (Figures 4B–E).

**FIGURE 5:**
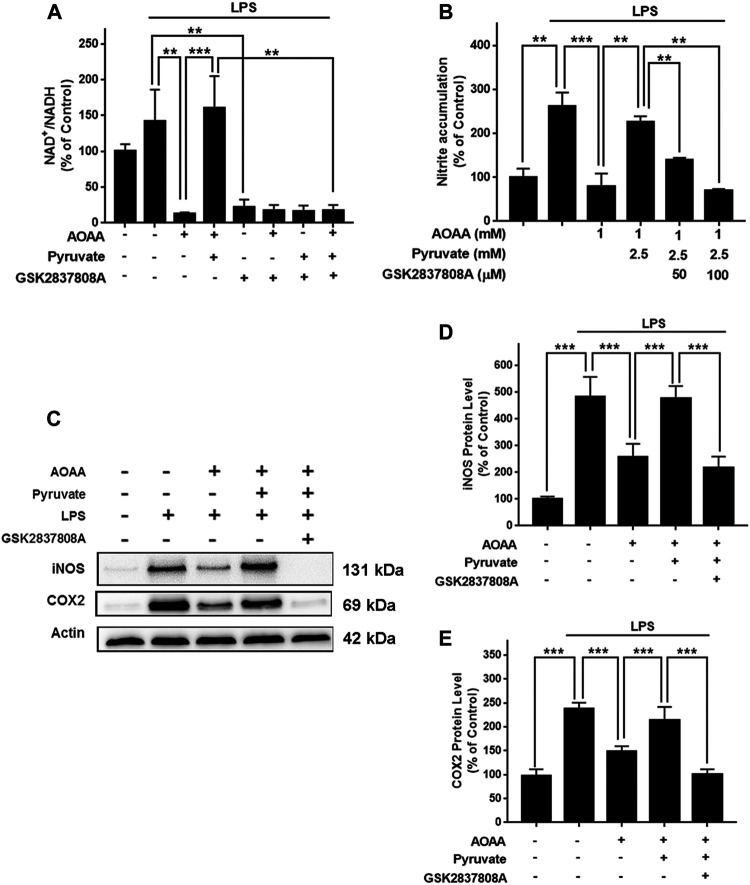
Pyruvate reversed the AOAA-produced decreases in the cytosolic NAD^+^/NADH ratio and microglial activation of LPS-treated BV2 microglia by promoting LDH-mediated consumption of cytosolic NADH. **(A)** Treatment of the cells with 100 μM GSK2837808A completely reversed the effects of pyruvate on the cytosolic NAD^+^/NADH ratio of the microglial cell cultures treated with LPS and AOAA. **(B)** Treatment of the cells with 50 and 100 μM GSK2837808A dose-dependently attenuated the effects of pyruvate on the LPS-induced NO production of the AOAA-treated microglia. **(C)** Western blot assays showed that treatment of the cells with 100 μM GSK2837808A reversed the effects of pyruvate on the protein levels of iNOS and COX2. **(D)** Quantifications of the Western blot assays showed that treatment of the cells with 100 μM GSK2837808A significantly attenuated the effects of pyruvate on the protein levels of iNOS. **(E)** Quantifications of the Western blot assays showed that treatment of the cells with 100 μM GSK2837808A significantly attenuated the effects of pyruvate on the protein levels of COX2. After pre-treatment with 1 mM AOAA for 30 min, the BV2 cells were incubated with 2.5 mM pyruvate, 1 µg/ml LPS, and 50 or 100 µM GSK2837808A for 20 h (for NAD^+^/NADH assay) or 23.5 h (for NO, iNOS, and COX2 assays). *N* = 3. The data were collected from three independent experiments. **, *p* < 0.01; ***, *p* < 0.001.

We applied GSK2837808A, an inhibitor of LDH, to test out hypothesis that pyruvate reversed the AOAA-produced decreases in the cytosolic NAD^+^/NADH ratio of LPS-treated BV2 microglia by promoting LDH-based consumption of cytosolic NADH. We found that treatment of the cells with 100 μM GSK2837808A completely reversed the effects of pyruvate on the cytosolic NAD^+^/NADH ratio of the microglia treated with LPS and AOAA (Supplementary Figure 3F and Figure 5A). Moreover, treatment of the cells with 50 and 100 μM GSK2837808A dose-dependently attenuated the effects of pyruvate on the LPS-induced NO production of the AOAA-treated microglia (Figure 5B); and treatment of the cells with 100 μM GSK2837808A reversed the effects of pyruvate on the protein levels of iNOS and COX2 of the LPS- and AOAA-treated microglia (Figures 5C–E). The reagents at the concentrations used in our study did not significantly affect the survival of BV2 microglia (Supplementary Figure 3G).

### MAS Mediates LPS-Induced Microglial Activation by Regulating STAT3 Phosphorylation

**FIGURE 6:**
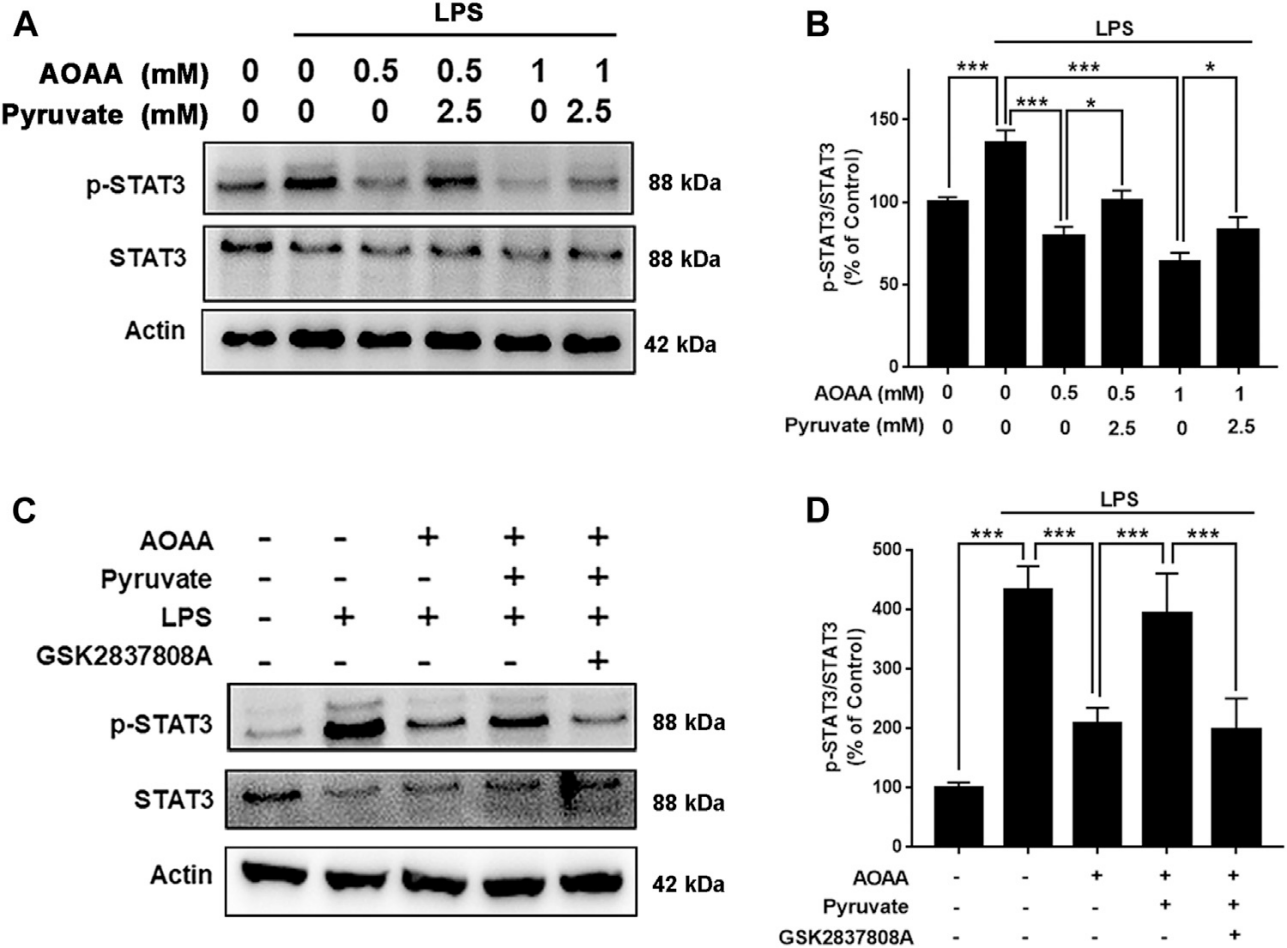
MAS mediates LPS-induced microglial activation by regulating STAT3 phosphorylation. **(A, B)** Both 0.5 and 1 mM AOAA significantly decreased phosphorylation of STAT3 in the LPS-treated microglia, which was reversed by pyruvate treatment. **(C, D)** The LDH inhibitor GSK2837808A abolished the effect of pyruvate on STAT3 phosphorylation of the microglia treated with LPS and AOAA. After pre-treatment with 1 mM AOAA for 30 min, the BV2 cells were incubated with 2.5 mM pyruvate, 100 µM GSK2837808A, and 1 µg/ml LPS. 2 h after the incubation, Western blot assays were conducted. *N* = 3. The data were collected from three independent experiments. *, *p* < 0.05; ***, *p* < 0.001.

Since STAT3 phosphorylation plays a significant role in inflammation, including LPS-induced neuroinflammation (Qin et al., 2016; Qin et al., 2017; Hu et al., 2019; Yang et al., 2019), we tested our hypothesis that MAS mediates LPS-induced microglial activation by regulating STAT3 phosphorylation. We found that both 0.5 and 1 mM AOAA significantly decreased phosphorylation of STAT3 in the LPS-treated microglia, which was reversed by pyruvate treatment (Figures 6A,B). The LDH inhibitor GSK2837808A also abolished the effect of pyruvate on STAT3 phosphorylation of the microglia treated with LPS and AOAA (Figures 6C,D).

## Discussion

NAD^+^/NADH metabolism plays crucial roles in both aging and numerous diseases (Ying 2008; Verdin 2015). A previous study reported that metformin-produced decreases in NAD^+^/NADH ratio led to suppression of cancer cell proliferation (Gui et al., 2016). However, there has been significant deficiency in the information regarding the roles of NAD^+^/NADH metabolism in inflammation. Since inflammation is a key pathological factor in numerous diseases, it is warranted to elucidate the relationships between NAD^+^/NADH metabolism and inflammation. Our current study has provided several lines of evidence indicating that MAS is a novel mediator of LPS-induced neuroinflammation: AOAA produced profound decreases in both cytosolic NAD^+^/NADH ratios and activation of LPS-treated BV2 microglia, both of which were reversed by pyruvate. Moreover, the LDH inhibitor GSK2837808A blocked the effects of pyruvate on the AOAA-produced decreases in the cytosolic NAD^+^/NADH ratio as well as the LPS-induced microglial activation.

Our current finding regarding the critical role of MAS in LPS-induced neuroinflammation has provided a novel mechanism for elucidating the role of MAS in multiple neurodegenerative diseases. Our findings have also indicated a new mechanism underlying the roles of NAD^+^/NADH metabolism in multiple neurodegenerative diseases. Moreover, our finding may provide valuable information for elucidating the mechanisms underlying the beneficial effects of NMN, a NAD^+^ precursor, on the pathological process (Mills et al., 2016; Yoshino et al., 2018; Kiss et al., 2019). Future studies are warranted to further investigate the relationships among MAS, cytosolic NAD^+^/NADH ratio, neuroinflammation, and multiple neurological disorders.

STAT is a cellular signal transcription factor involved in the regulation of many cellular activities, such as cell differentiation and proliferation in normal cells (Hillmer et al., 2016). It has been found that STAT3 plays an important role in the inflammatory responses (Benveniste et al., 2012; Qin et al., 2016). Our current study has investigated the mechanisms underlying the role of MAS in neuroinflammation, indicating that MAS inhibition leads to decreased LPS-induced neuroinflammation at least partially by inhibiting STAT3: AOAA significantly inhibited LPS-induced phosphorylation of STAT3, which was reversed by pyruvate treatment. Moreover, the effects of pyruvate on the LPS-induced activation of AOAA-treated microglia were also reversed by the LDH inhibitor. It has been reported thatSTAT3 transactivated MDH1 to sustain MAS activity and the self-renewal and differentiation of hematopoietic stem cells (Gu et al., 2020). These findings have collectively indicated the interactions between STAT3 and MAS.

It is noteworthy that MAS activity significantly affect not only the cytosolic NADH levels, but also the levels of the multiple important metabolites including glutamate, aspartate, oxaloacetate, malate and α-ketoglutarate (Borst 2020). Therefore, our finding indicating an important role of MAS in LPS-induced microglial activation does not indicates directly that the MAS-produced effects on the cytosolic NADH level is the sole factor that mediates the effects of MAS on LPS-induced microglial activation: It remains possible that the MAS-mediated changes of the levels of the metabolites and cytosolic NADH levels may play synergistic effects on LPS-induced microglial activation. Future studies are warranted to investigate the potential roles of the other metabolites in the AOAA-produced inhibition of LPS-induced microglial activation. These synergistic effects may account for the seemingly contradictions between our study and a previous report (Shen et al., 2017). It is noteworthy that there is a profound difference between the model of LPS-induced microglial activation used in our study and that used in the previous report (Shen et al., 2017): The LPS concentration used in our study was 1 μg/ml, while the LPS concentration used in the previous report was 100 times lower - 10 ng/ml. This dramatic difference in the LPS concentration may produce profound differences in the mechanisms underlying the LPS-induced microglial activation, as reported by other studies (Ryu et al., 2019). Therefore, the dramatic differences in the LPS concentrations used in these two studies may be an important factor leading to different results from these two studies.

Our study has found that AOAA administration can produce profound inhibition of LPS-induced neuroinflammation of mice, as indicated by the inhibitory effects of AOAA on several indices of neuroinflammation in the LPS-treated mice: AOAA administration profoundly attenuated the LPS-induced increases in the Iba-1 levels, the protein levels of iNOS and COX2, as well as the mRNA levels of IL-1β, IL-6 and TNF-α. Collectively, our study has indicated that AOAA is a novel and effective drug for inhibiting LPS-induced neuroinflammation. Moreover, based on our findings indicating that AOAA produces its effects on LPS-induced neuroinflammation mainly by its effects on MAS, our study has also indicated that MAS is a novel target for modulating neuroinflammation in numerous neurological disorders. It is noteworthy that AOAA post-treatment can also produce profound inhibitory effects on LPS-induced microglial activation, which has indicated great potential for the therapeutic applications of the molecules for inhibiting neuroinflammation. This finding has also further highlighted the significance of MAS as a therapeutic target for attenuating neuroinflammation. Moreover, the findings from our study have provided a new piece of information highlighting a critical role of NAD^+^/NADH metabolism in inflammation.

AOAA has been widely used as an inhibitor of MAS in a number of studies: AOAA can inhibit post-infarct cardiac dysfunction by balancing macrophage polarization through modulating macrophage metabolism in mice (Zhao et al., 2020); and AOAA preserved mitochondrial functions after ischemia–reperfusion (Jespersen et al., 2017). However, there have also been studies indicating that AOAA can act as an inhibitor of cystathionine β-synthetase to produce anti-inflammatory effects in several animal models (Qu et al., 2013; Zhao et al., 2017; Damba et al., 2019; Spalloni et al., 2019). Considering that AOAA may also produce its effects by mechanisms other than MAS inhibition, we conducted studies to provide two lines of evidence indicating that AOAA produced its effects mainly by affecting MAS: First, pyruvate treatment reversed the effects of AOAA on the cytosolic NAD^+^/NADH ratio, which also restored the LPS-induced activation of the AOAA-treated microglia; and second, the LDH inhibitor GSK2837808A blocked the effects of pyruvate on the AOAA-produced decreases in both the cytosolic NAD^+^/NADH ratio and LPS-induced microglial activation.

LPS-induced neuroinflammation is a most widely used model of neuroinflammation (Batista et al., 2019). However, it is noteworthy that there are still significant differences between the LPS-induced neuroinflammatory responses and the neuroinflammatory responses in the neurological disorders such as AD, PD and cerebral ischemia. It is warranted to further determine if the brain damage of these neurological disorders may be attenuated by MAS inhibition.

## Conclusion

The major findings of our study include: First, both pre-treatment and post-treatment of AOAA profoundly decreased the LPS-induced neuroinflammation in mice. Second, pyruvate treatment reversed AOAA-produced decreases in both the cytosolic NAD^+^/NADH ratios and activation of LPS-treated microglia. Third, the LDH inhibitor GSK2837808A blocked the effects of pyruvate on the AOAA-produced decreases in both the cytosolic NAD^+^/NADH ratio and LPS-induced microglial activation, suggesting that pyruvate-promoted consumption of cytosolic NADH mediates the effects of pyruvate on the LPS-induced activation of the AOAA-treated microglia. Fourth, AOAA produced inhibition of LPS-produced microglial activation at least partially by decreasing STAT3 phosphorylation. Collectively, our findings have provided evidence not only indicating MAS as a novel mediator in LPS-induced neuroinflammation, but also indicating AOAA as a new and effective inhibitor of LPS-induced neuroinflammation.

## Data Availability

The original contributions presented in the study are included in the article/Supplementary Material, further inquiries can be directed to the corresponding author.

## Supplementary Material

Supplementary Figure 1 Immunofluorescence staining assays indicated that both AOAA pre-treatment and post-treatment attenuated the LPS-induced increases in the protein levels of COX2 in the hippocampus and the cerebral cortex of mice. **(A)** The immunofluorescence staining assay showed that both pre-treatment and post-treatment of AOAA decreased LPS-induced COX2 increases in the hippocampus of the mice. **(B)** The immunofluorescence staining assay showed that both pre-treatment and post-treatment of AOAA decreased LPS-induced COX2 increases in the cerebral cortex of the mice. **(C)** Quantifications of the images indicated that both pre-treatment and post-treatment of AOAA significantly decreased LPS-induced COX2 increases in the hippocampus of the mice. **(D)** Quantifications of the images indicated that both pre-treatment and post-treatment of AOAA significantly decreased LPS-induced COX2 increases in the cerebral cortex of the mice. The mice were administered with LPS (250 μg/kg, i.p,). AOAA was administered 3 days before LPS administration or 3 days after LPS administration. 7 days after the LPS administration, the immunofluorescence staining assays were conducted. Scale bar = 100 μm. *N* = 5. *, p < 0.05; **, p < 0.01; ***, p < 0.001.

Supplementary Figure 2 The effects of saline, AOAA and LPS on the body weight of mice. **(A)** Administration (i.p) of saline did not significantly affect the body weight of mice. **(B)** Administration (i.p) of AOAA did not significantly affect the body weight of mice. **(C)** Administration (i.p) of LPS (250 μg/kg) led to significant decreases in the body weight of mice. **(D, E)** Co-administration (i.p) of AOAA and LPS led to decreases in the body weight of mice. AOAA was administered either 3 days before LPS administration or 3 days after LPS administration. 7 days after LPS administration, the mice were sacrificed. *N* = 10.

Supplementary Figure 3 The effects of various concentrations of LPS, AOAA, pyruvate and GSK2837808A on the nitrite accumulation and cell survival of BV2 microglia. **(A)** LPS treatment dose-dependently increased nitrite accumulation in BV2 microglia. The cells were treated with 0.1, 0.5, 1, 2 or 10 μg/ml LPS for 24 h. **(B)** AOAA pre-treatment dose-dependently inhibited LPS-induced nitrite accumulation in BV2 microglia. After pre-treatment of the cells with 0.1, 0.5, 1, 2 or 10 mM AOAA for 30 min, the BV2 cells were incubated with 1 µg/ml LPS for 23.5 h. **(C)** AOAA pre-treatment led to significant decreases in the survival of BV2 microglia. After pre-treatment of the cells with 0.1, 0.5, 1, 2 or 10 mM AOAA for 30 min, the BV2 cells were incubated with 1 µg/ml LPS for 23.5 h. **(D)** Pyruvate at the concentration of 2.5 mM completely prevented AOAA-produced nitrite accumulation in BV2 microglia. After pre-treatment of the cells with 1 mM AOAA for 30 min, the BV2 cells were incubated with and 1 µg/ml LPS and 1, 2.5 or10 mM pyruvate for 23.5 h. **(E)** Co-administration of the cells with AOAA, LPS and pyruvate did not significantly affect the survival of BV2 microglia. After pre-treatment with 1 mM AOAA for 30 min, the BV2 cells were incubated with 1 µg/ml LPS and 1, 2.5 or 10 mM pyruvate for 23.5 h. **(F)** Treatment of the cells with GSK2837808A dose-dependently attenuated the effect of pyruvate on AOAA. After pre-treatment of the cells with 1 mM AOAA for 30 min, the BV2 cells were incubated with 2.5 mM pyruvate, 1 µg/ml LPS and GSK2837808A (50, 100 or 200 µM) for 23.5 h. **(G)** Co-treatment of the cells with 1 mM AOAA, 2.5 mM pyruvate, 1 µg/ml LPS and 100 µM GSK2837808A did not affect the survival of BV2 cells. After pre-treatment of the cells with 1 mM AOAA for 30 min, the BV2 cells were incubated with 2.5 mM pyruvate, 1 µg/ml LPS and 100 µM GSK2837808A for 23.5 h. *N* = 3. The data were collected from three independent experiments. *, *p* < 0.05; **, *p* < 0.01; ***, *p* < 0.001.
