# Supplementary figures and images for: Malate-Aspartate Shuttle Plays an Important Role in LPS-Induced Neuroinflammation of Mice Due to its Effect on STAT3 Phosphorylation
Source: Front Mol Biosci. 2021 Jul 26;8:655687. doi: 10.3389/fmolb.2021.655687 (PMC8350486; doi:10.3389/fmolb.2021.655687)

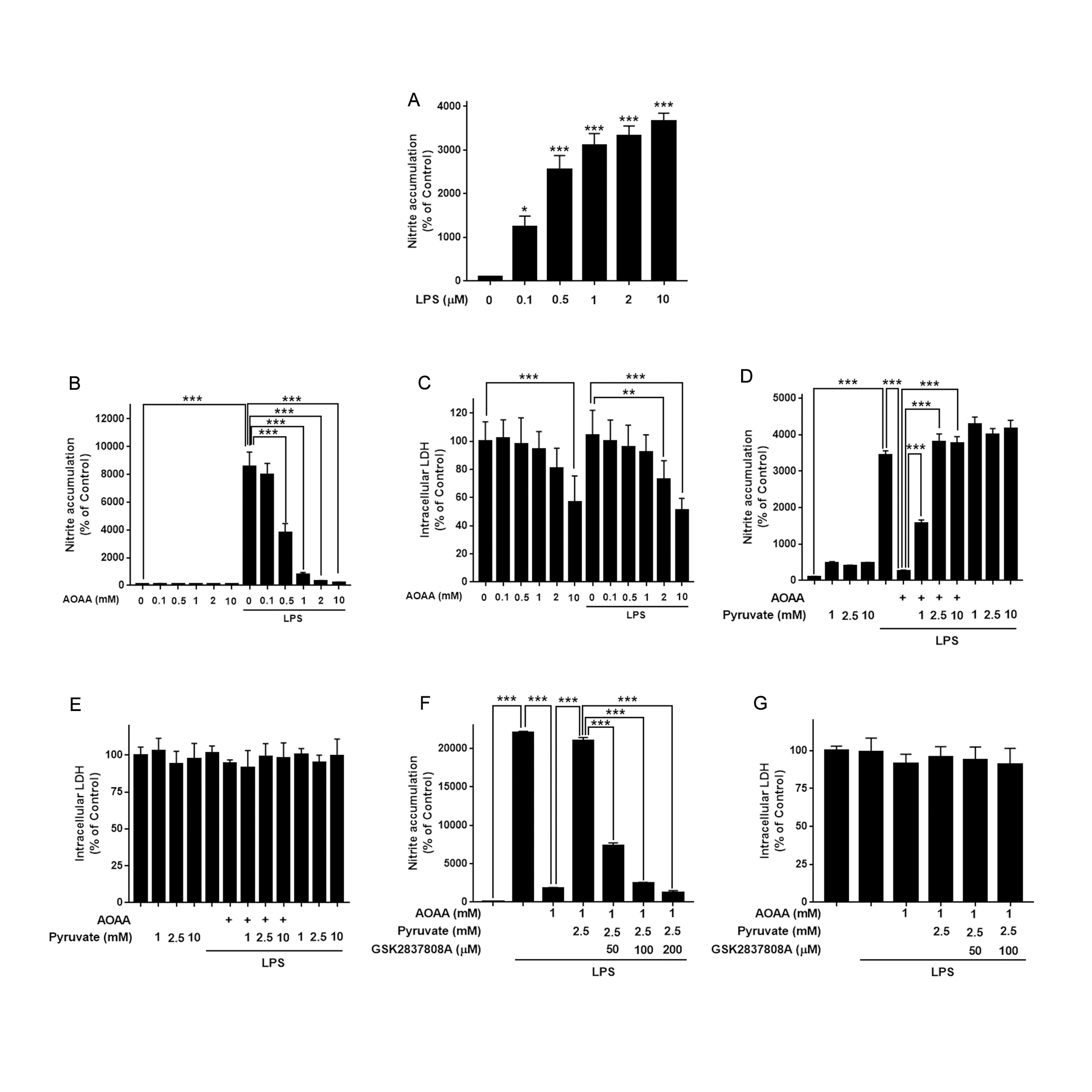

Supplement: Supplementary file 1 [file Image3.TIF]

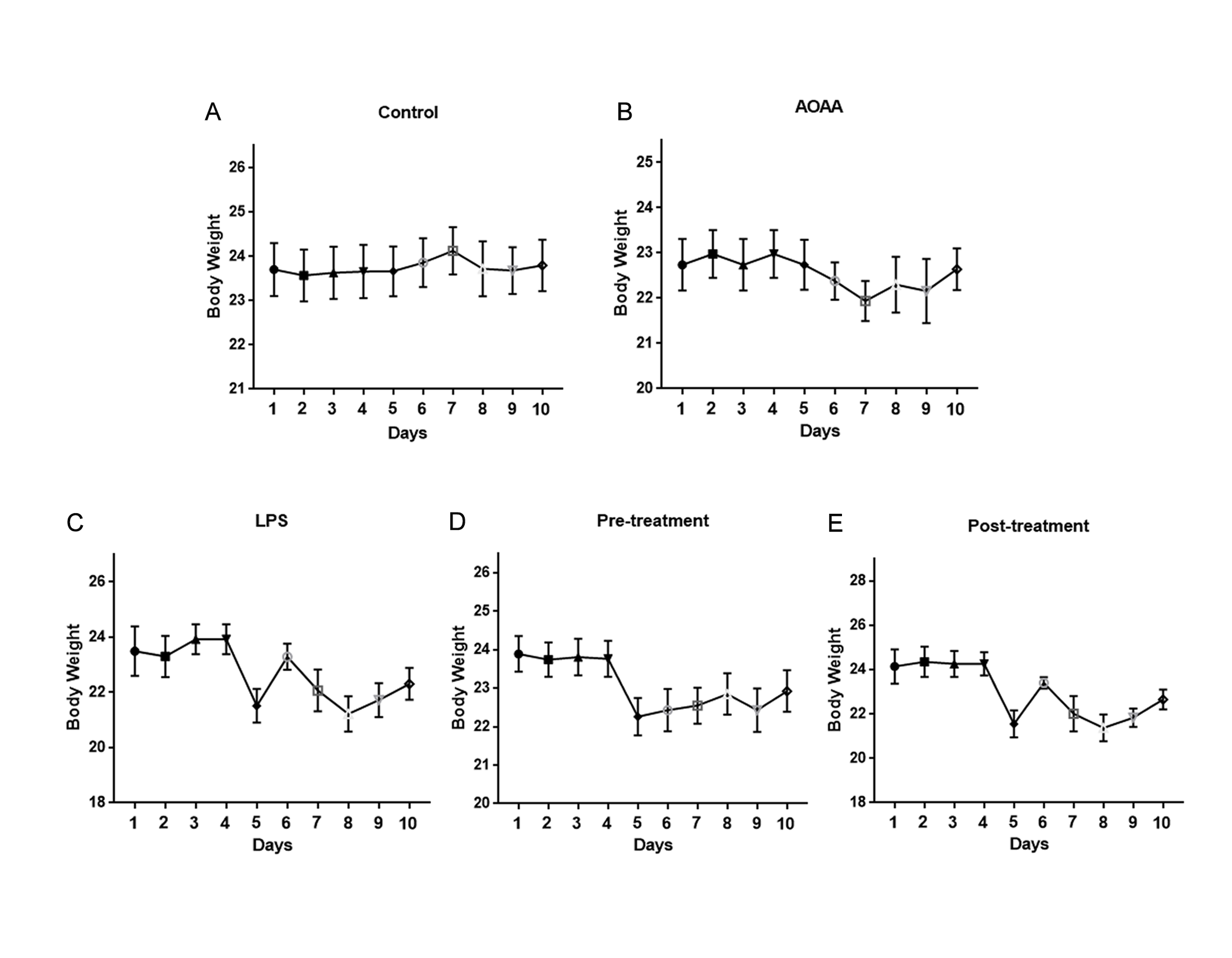

Supplement: Supplementary file 2 [file Image2.TIF]

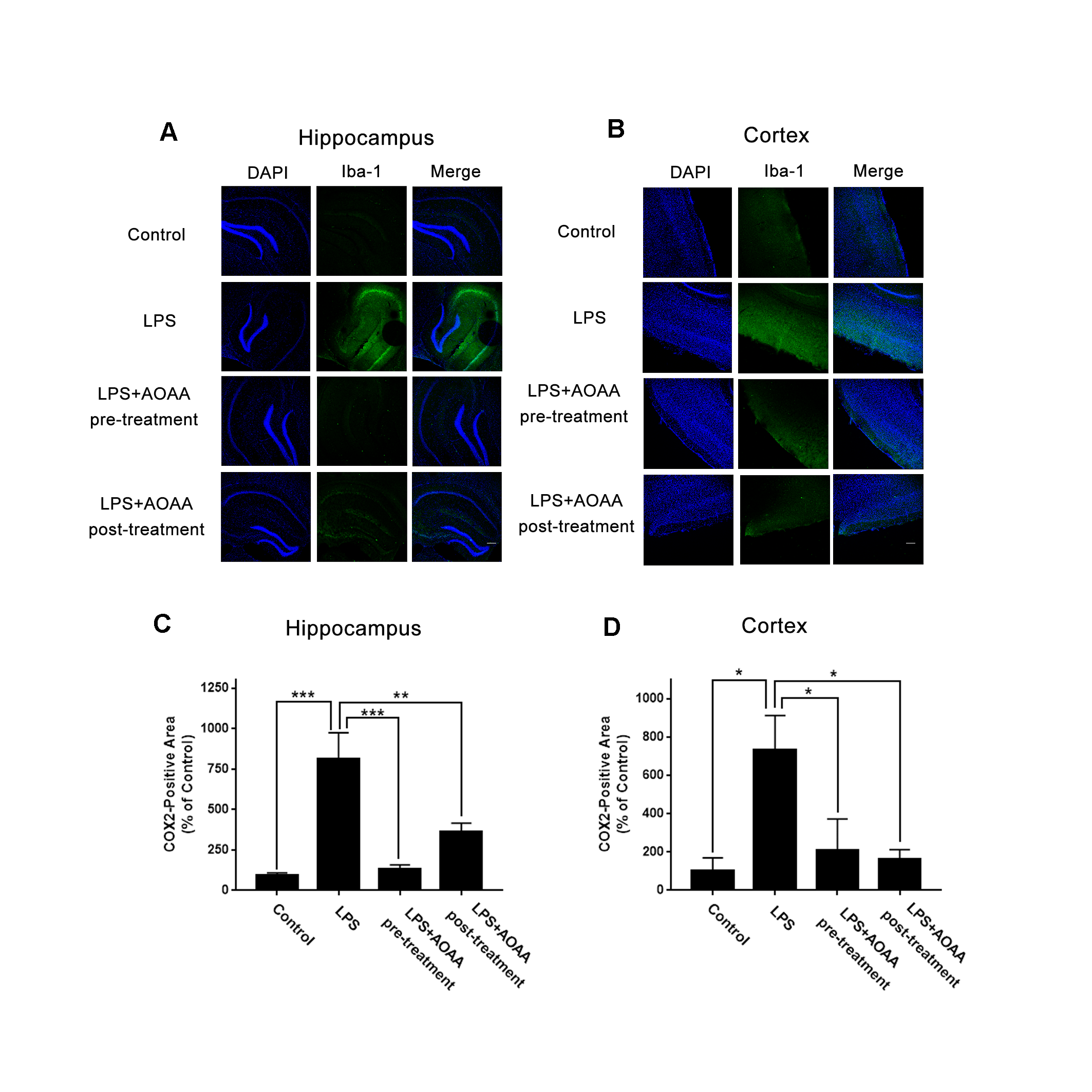

Supplement: Supplementary file 3 [file Image1.TIF]
